# 'Damage control orthopaedics' in patients with delayed referral to a tertiary care center: experience from a place where Composite Trauma Centers do not exist

**DOI:** 10.1186/1752-2897-2-2

**Published:** 2008-01-29

**Authors:** Shabir Ahmed Dhar, Masood Iqbal Bhat, Ajaz Mustafa, Mohammed Ramzan Mir, Mohammed Farooq Butt, Manzoor Ahmed Halwai, Amin Tabish, Murtaza Asif Ali, Arshiya Hamid

**Affiliations:** 1 Department of Orthopaedics, Government Medical College, Srinagar, Jammu and Kashmir, India; 2 Department of Surgery, Sheri Kashmir institute of medical sciences, Srinagar, Jammu and Kashmir, India; 3 Department of Hospital Administration, Sheri Kashmir institute of medical sciences, Srinagar, Jammu and Kashmir, India; 4 Department of Anaesthesia and critical care, Sheri Kashmir institute of medical sciences, Srinagar, Jammu and Kashmir, India

## Abstract

**Background:**

Management of orthopaedic injuries in polytrauma cases continues to challenge the orthopaedic traumatologist. Mass disasters compound this challenge further due to delayed referral. Recently there has been increasing evidence showing that damage control surgery has advantages that are absent in the early total care modality. We studied the damage control modality in the management of polytrauma cases with orthopaedic injuries who had been referred to our hospital after more than 24 hours of sustaining their injuries in an earthquake. This study was conducted on 51 cases after reviewing their records and complete management one year after the trauma.

**Results:**

At one year, out of the 62 fractures, 3 were still under treatment, while the others had united. As per the radiological and functional scoring there were 20 excellent, 29 good, 5 fair and 5 poor results. In spite of the delayed referral there was no mortality.

**Conclusion:**

In situations of delayed referral in areas where composite trauma centers do not exist the damage control modality provides an acceptable method of treatment in the management of polytrauma cases.

## Background

On 8^th ^October 2005, at 9:20 am IST, an earthquake of magnitude 7.8 on Richter scale struck the Kashmir region of Asia. Around 90,000 people died in this natural disaster.

In view of the massive morbidity arising out of this event, the centrally located hospitals in the Srinagar city 120 kilometers away from the mainly affected areas, received 823 patients over a period of 5 days. A significant number of these patients had sustained polytrauma with involvement of multiple systems, in addition to long bone and pelvic fractures.

The principles of fracture management in poly-trauma patients continue to be of crucial importance. Over the last few decades various strategies of fracture treatment in the multiply injured have evolved [1]. The concept of early total care (ETC) developed in the 80s. Later it became apparent that certain patients did not benefit from ETC. Indeed adverse outcome was encountered. In spite of this, delaying all orthopedic surgery is also, not always the best approach [2]. In such situations the principle of Damage Control Orthopedics (DCO) may be used. According to Katsoulis et al the DCO principle should be applied for skeletal stabilization in patients of poly trauma, the intent being to allow immediate fracture fixation in patients who are not cleared for definitive fracture care [3,4].

In mass disasters, orthopaedic care possesses special challenges. Not only are the wounds contaminated but the patients have to undergo prolonged evacuation and staged resuscitation which complicates the basic injuries.

The principle of this study is to study the efficacy of damage control orthopaedics, when applied to 51 cases of polytrauma in a mass disaster setting in a situation where composite trauma centers do not exist. The study documents the advantages of applying damage control orthopaedics in a mass disaster where enormous patient loads are encountered and hospital resources are stretched to the limit. The study also documents the complications associated with such a treatment modality.

## Patients and methods

This study was compiled retrospectively with the data of the poly trauma cases admitted to the Government Bone and Joint Surgery Hospital Srinagar 24 hours after the earthquake of October 8^th ^2005. 528 cases (468 + 60) were received by two main orthopaedic specialty hospitals located around a hundred kilometers from the site of involvement. The patients who arrived and were managed within 24 hours were excluded from the retrospective analysis to focus on the patients with delayed referral. 51 patients fitted the inclusion criteria i.e direct referral from the site, new injury severity scores more than 18, involvement of more than 2 systems. Only patients with at least one of the following 4 fractures were included.

1. Femoral fractures

2. Tibial fractures.

3. Unstable pelvic ring fractures.

4. Compound fractures of the humerus.

The injury severity score of these patients was calculated on reviewing the final records. The fractures were classified as per the OTA classification [5]. Compound injuries were classified as per the Gustilo and Anderson classification [6]. Closed soft tissue injuries were classified as per the classification given by Tscherne and Gotzen [7].

All 51 cases were initially managed with external fixation. Unstable metaphyseal and intra-articular fractures were fixed transarticularly.

The open wounds were managed by primary debridement, pulsed lavage and drainage. None of the wounds was closed primarily in view of the extent of contamination and delayed referral.

After fracture stabilization the patients were referred for the management of neurosurgical, cardiovascular, thoracic, plastic and general surgery consultation to the nearest hospitals with availability of super-specialty in these modalities. The pin sites were dressed daily with a mixture of dilute hydrogen peroxide and povidone iodine.

The patients were referred back after relevant interventions in these hospitals for the definitive management of the orthopaedic injuries. Conversion to definitive fixation was performed when the platelet count was above 100,000/μl and PO2/FIO2 ratio >280.

**Figure 1:**
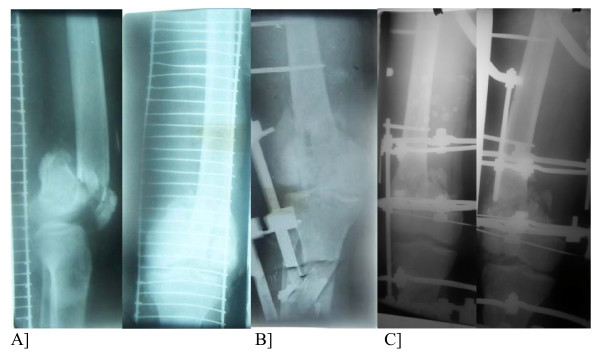
A]Compound Intraarticular fracture of the distal femur. B] Stabilized by a transarticular external fixator. C] Final conversion to the Ilizarov fixator.

**Figure 2:**
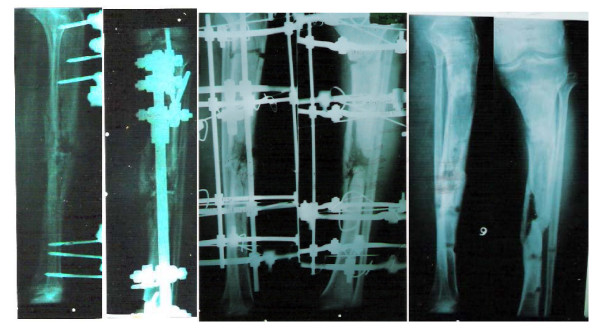
Type II Compound fracture tibia converted to an Ilizarov fixator, and the final result.

**Figure 3:**
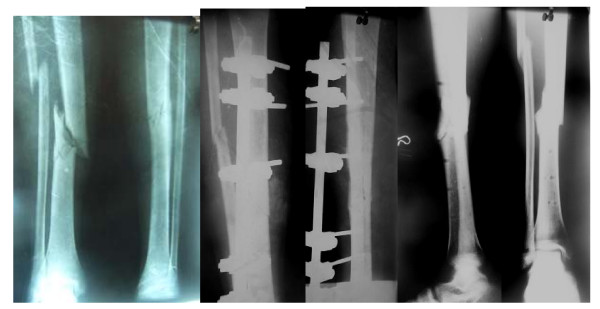
Type IIIa fracture tibia stabilized by an external fixator. The fracture showed signs of union on return referral whence this treatment modality was continued. The final result shows union.

**Figure 4:**
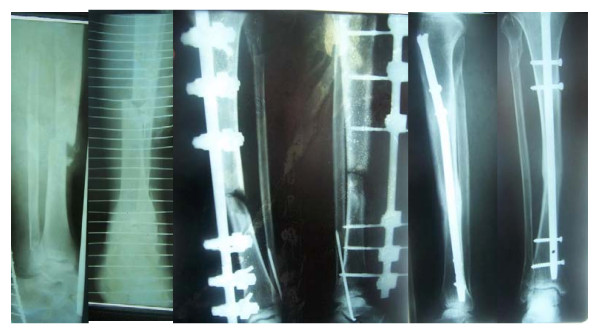
Type IIIb fracture of the tibia stabilized by an external fixator. The fracture was converted to an interlocking nail and the final result is shown.

The patients were classified into two groups i.e. infected as defined by drainage from the wound and pin sites and non infected where the aforementioned signs were absent. All infected cases were managed with the Ilizarov methodology. Closed fractures were managed by conversion to Intramedullary nailing if the fracture location was diaphyseal. Cases where reverse referral was delayed for more than 4 weeks were reassessed in terms of the reduction and formation of a callus. In case the condition was satisfactory the callus was allowed to consolidate with the fixator in situ. All 51 patients were followed up for 1 year and their records assessed at one year in terms of number of interventions, radiological union, function and complications. The bone results which were assessed according to the protocol laid down by the association for the study and application of the method of Ilizarov[8,9] An excellent result was defined as union, no infection, deformity of less than 7° and leg length inequality of less than 2.5 cm; a good result was defined as union and any two of the other three criteria; a fair result was defined as union and one of the other criteria; and a poor result was defined as non-union or refracture, or as union in the absence of any of the other three criteria. The functional result was calculated as per the Ilizarov criteria[9]. A noteworthy limp, stiffness of adjacent joints (loss of more than 15° of motion), soft tissue sympathetic dystrophy (RSOD), pain that reduced activity or disturbed sleep and inactivity. The functional result was considered excellent if the patient was active and none of the other four criteria were applicable, good if the patient was active but one or two of the other criteria were applicable, fair if the patient was active but three or four of the other criteria were applicable and poor if the patient was inactive. Figures 1, 2, 3, 4.

## Results [See Additional file 1]

Of the 526 cases who were referred to Orthopaedic units in the city, 51 had a new injury severity score NISS above 18. These patients had been referred from the quake hit areas 24 hours after receiving their injuries. This group of patients with multiple injuries in addition to orthopaedic injuries had an average age of 29.68 years (15–71 years) with a male to female ratio of 29:22. Accompanying injuries were thoracic injuries, brain injuries, abdominal trauma and vascular injuries in 15, 22, 25 and 13 patients respectively. 52.9% of our patients had deranged kidney function, a sequel of the crushing trauma and delayed referral. 8 patients went on to develop renal failure in spite of renal protective protocols. 6 patients were managed by the DCO modality on day 2, 24 on day 3, 12 on day 4 and 9 on day 5. The average NISS of these patients was 23. There were 31 tibial fractures, 27 femoral fractures, 9 humeral fractures and 5 unstable pelvic fractures. According to the OTA classification there were 27 linear, 16 comminuted, 2 segmental and 11 cases with bone loss. 20 fractures were closed with 5 being G I and 15 G II as per the Tsherne and Gotzen classification. Of the 37 open fractures there were 9 type 1, 21 type II and 8 type III fractures as per the Gustilo and Anderson classification. All fractures were fixed using the AO fixators. 12 were used transarticularly in addition to the 5 pelvic fixators. All patients were referred for further management on the day of their operation for super-specialty management of their injuries. 36 additional procedures were carried out on 26 patients in these centers. 31 patients required intensive care monitoring. The average duration of return referral and surgery to the orthopaedic units was 21.5 days (7–61 days). 9 fractures were treated conservatively, 23 by interlocking nailing, 1 by plating and 28 were converted to the Ilizarov ring fixator.

At one year, out of the 62 fractures, 3 were still under treatment, while the others had united. As per the radiological and functional scoring there were 20 excellent, 29 good, 5 fair and 5 poor results. In spite of the delayed referral there was no mortality.

Complications at one year included 3 persistent non unions, 4 cases with significant stiffness and two cases with infection with both having osteomyelitis.

## Discussion

Early fixation of fractures has been found to significantly reduce the incidence of pulmonary complications and organ failure and to improve survival [10,11]. The principles of fracture management in polytrauma patients continue to be of crucial importance. Over the last 5 decades various strategies of fracture treatment in the multiply injured have been evolved. The various new methodologies remain controversial [1].

The concept of total care (ETC) developed in the 80s with O' Brien et al stating that in a majority of cases of femoral shaft fracture, interlocking, intramedullary nailing can be done.

Oztuna et al found in their experimental study that early internal fixation of long bones results in decreased bilateral translocation from the gut [12]. Complications of fractures have been noted for many decades. When treated non operatively in traction, approximately 20% of young people with femur fractures would develop some manifestations of fat embolism. Riska et al also observed that early fixation of femoral fractures resulted in a drop in the fat embolism syndrome [13]. Several studies have documented the reduction in pulmonary complications and organ failure in early fracture fixation [10,11,14,15].

Recently however application of early total care has been reported as not being beneficial to all the patients, with adverse outcome being encountered in poly trauma patients [1] The application of early total care in cases with co existing chest injuries, head trauma and those with mangles extremities may be potentially harmful [2]. There is also evidence that an increased complication rate may be encountered in such cases [6,16].

Pape et al in their study of 35 patients found a sustained inflammatory response after intramedullary instrumentation. Reinforcing the clinical importance of this, they named it as the phenomenon of the second hit [12]. Additional operative trauma may cause an inflammatory body reaction similar to the systemic reaction after mild to moderate accidental injury. [ISS < 25] Accordingly initial operative surgery exceeding 6 hours is critical for the outcome [17].

Border stated that the realization that problems that cause death later on, or produce major problems in ICU care, begin with resuscitation and are present only in those with severe injuries. He also attributed the difficulty of doing the femoral fractures the night of the admission with severe chest injuries, not with the intramedullary nail, but with the reaming [18]. The correct treatment of an injured extremity involves understanding the entire reconstruction process, post operative management and rehabilitation. It is therefore important that the initial stabilization includes the vision of definitive fracture care [19]. Performance of limited surgical interventions subsequently reduces blood loss and transfusion requirements. This can only be beneficial in these critically ill patients, reducing the risk of developing systemic complications and early mortality [20]. The principle of Damage Control orthopaedics (DCO) was used for the first time by Orthopaedic Surgeons from R. Adams Cowley Shock Trauma Center [3]. The intent of this principle is not to postpone fracture stabilization but to allow immediate fracture fixation in patients who are not cleared for definitive fracture care.

Orthopedic Management of a large number of polytrauma cases in a setting of mass disaster with its inherent challenges has never been studied. The fracture care in polytrauma cases in mass disasters is complicated further by the occurrence of crush syndrome, renal failure, contamination and neurovascular compromise [21].

Covey documented the difficulties encountered in managing mass casualties. The challenges require the patients to be triaged and treated in an austere and dangerous environment, undergo staged resuscitation and definitive surgery and endure prolonged evacuation, often involving air and ground transport [22].

In situations of polytrauma with delayed referral being the norm rather than the exception, the cases are at a higher risk from the second hit. Damage control orthopedics in such situations may provide additional advantage that might manifest in terms of better overall care of patients. This includes lower requirement of blood transfusions and reduced operating time.

External fixations as a prime modality for the application of damage control Orthopedics provide the following advantages:

• Decreased operating time

• Decreased blood loss

• Does not increase local complications

• Quality of definite osteosynthesis is not impaired

The soft tissue injuries and associated wound contamination is so severe that in these cases the pin sites do not represent a significant additional source for infection. The small bacterial inoculum inherent to the pin sites is often not sufficient to overcome host defenses to cause deep septic complications, even in the presence of physiologic complications which are accentuated by delayed referral. In all our cases which were converted to intramedullary nails, excision of pin tracts with wash out was done. The fixators provided more than adequate stabilization to facilitate nursing and eliminated fracture movement. The fixators also allowed good wound care and physiotherapy.

Our series was complicated by the coexistence of three problems in combination i.e. injuries sustained in a mass disaster, polytrauma and delayed referral. In such a situation application of damage control orthopaedics is not only a reasonable alternative but perhaps the most judicious one as well.

Delayed referral complicates management of polytrauma cases. Hirschberg et al mentioned high observed rates of multiple organ failure in patients surviving the initial 24 hours after their injuries [23]. These processes seem to be initiated by cascading events resulting from blood loss and inflammatory release leading to a 'vicious circle' of shock, hypothermia, acidosis and coagulopathy resulting in end organ failure [24]. Delayed referral from the contaminated and austere surroundings of a mass disaster means that the patient has already sustained a 'second hit' in terms of the delay. It is difficult for any of the injury scoring systems to justifiably grade the patients, and hence the criteria for application of damage control might vary. An early definitive surgery in such situations might equal a 'third hit'.

Mass disasters tend to overwhelm the capacity of the hospitals to cope with the massive and relatively unexpected load of patients. This situation often overwhelms the surge capacity of the hospitals as well. In such situations, to facilitate the care of polytrauma patients as well as the patients with lesser injuries, the management of operating time takes prime importance. The average time taken to attain the preliminary fixation in our patients was 38.5 minutes. In the absence of any comparable study we found this time to be 37% of the time taken to fix a similar series of fractures dealt with a total care methodology in our hospital. This represents a significant saving in the operating time and a judicious use of stretched theatre resources.

Even though predictive factors for the individual trauma patient that would allow identification of patients who are too ill to undergo early total care are still lacking, the injury severity systems constitute reasonable predictors of potential complications [25].

Our study is unique in several respects. It reports a one year followup of polytrauma cases referred after a delay from the site of trauma. The damage control method was applied in the absence of a composite trauma center and all cases required referral to nearby hospitals for specialized management of other coexisting injuries. We applied the interlocking intramedullary nailing in cases where infection was absent and ring fixators in all cases with indicators of infection. We carried out conversion osteosynthesis by intramedullary nailing in cases which took up to 33 days for return referral. Only one case of deep infection was encountered which was dealt by early removal of hardware. Out of the 11 intraarticular fractures 4 developed stiffness after completion of treatment. We feel this group which necessitates trans articular fixation in the damage control mode might not benefit from this modality in the broad sense. Two of these were however managed by manipulation. 18 of our cases developed pin tract infections due to the external fixators applied before the final conversion. All of these were managed by debridement, excision and antibiotics.

This study is limited by the lack of a comparative study conducted elsewhere in similar circumstances.

## Conclusion

Application of damage control orthopaedics in mass disasters in situations where composite trauma centers do not exist appears to be a justifiable modality of management. The advantages it provides are

1. Reduced operating time. 2. Reduced blood loss. 3. Allows easy transport. 4. Conversion osteosynthesis is not impaired if the ring fixators are applied instead of the intramedullary nailing at the suspicion of infection. 5. Mortality is reduced. 6. Complications are minimal except in intraarticular fractures.

The DCO modality may be recommended in mass disasters due to these advantages which have significant ramifications in such situations.

## Competing interests

The author(s) declare that they have no competing interests.

## Authors' contributions

SAD is the principal author of this manuscript. MIB, AM, AT helped in the collection of the data. MRM, MFB, MAH helped in drafting the discussion. MAA, AH undertook the statistical analysis and read the final drafts.

## Supplementary Material

Additional file 1 Patient data of the 51 patients managed by damage control orthopaedics. Statistical data obtained from the 51 patients managed by damage control orthopaedics. Click here for file
