# Supplementary material for: 'Damage control orthopaedics' in patients with delayed referral to a tertiary care center: experience from a place where Composite Trauma Centers do not exist
Source: J Trauma Manag Outcomes. 2008 Jan 29;2:2. doi: 10.1186/1752-2897-2-2 (PMC2253507; doi:10.1186/1752-2897-2-2)
Supplement: Additional file 1 — Patient data of the 51 patients managed by damage control orthopaedics. Statistical data obtained from the 51 patients managed by damage control orthopaedics. [file 1752-2897-2-2-S1.doc]

ADDITIONAL FILE 1

SHOWING PATIENT DATA OF THE 51 PATIENTS MANAGED BY DAMAGE CONTROL ORTHOPAEDICS.

| **Sno.** | **Age**  **[ years]** | **Sex** | **Referral Delay**  **[ Days]** | **Fracture**  **[ Bone]** | **NISS** | **External Fixator** | **Additional surgery**  **[ Number]** | **Fracture type**  **[ OTA]** | **STI**  **[Gustilo Anderson]** | **Compound Injury**  **[Tscherne Gotzen]** | **Return Referral**  **[ Days]** | **Converted to** | **Status at 1 year** | **Complications.**  [ At one year] |
| --- | --- | --- | --- | --- | --- | --- | --- | --- | --- | --- | --- | --- | --- | --- |
| **1.** | **23** | **M** | **2** | **T, F** | **27** | **1** | **1** | **L,Co** | **II, II** |  | **17 days** | **ILN × 2** | **U,U** |  |
| **2.** | **27** | **F** | **2** | **F** | **27** | **TA** | **2** | **L** | **II** |  | **18 days** | **Illizarov** | **U,** | **Stiffness** |
| **3.** | **17** | **M** | **2** | **T** | **18** | **1** |  | **L** | **IIIa** |  | **14 days** | **ILN** | **U** |  |
| **4.** | **53** | **M** | **2** | **T** | **18** | **1** |  | **L** | **C** | **GII** | **14 days** | **ILN** | **U** |  |
| **5.** | **71** | **F** | **2** | **T** | **27** | **1** | **1** | **L** | **C** | **GII** | **19 days** | **ILN** | **U** |  |
| **6.** | **64** | **M** | **2** | **T** | **18** | **1** | **2** | **Co** | **C** | **GII** | **9 days** | **ILN** | **U** |  |
| **7.** | **19** | **M** | **3** | **T** | **27** | **TA** | | **Bl** | **IIIa** |  | **13 days** | **Ilizarov** | **U** | **Infection, Stiffness** |
| **8.** | **38** | **F** | **3** | **T** | **18** | **1** |  | **Bl** | **IIIb** |  | **27 days** | **Ilizarov** | **U** |  |
| **9.** | **23** | **F** | **3** | **F** | **18** | **TA** | **1** | **Bl** | **II** |  | **33 days** | **Ilizarov** | **U** |  |
| **10.** | **27** | **M** | **3** | **T** | **22** | **1** | **2** | **Co** | **C** | **GII** | **14 days** | **IL** | **U** |  |
| **11.** | **31** | **F** | **3** | **T** | **18** | **1** |  | **Co** | **C** | **GII** | **46 days** | **Con, Ilizarov** | **U** |  |
| **12.** | **43** | **F** | **3** | **T** | **22** | **1** | **1** | **L** | **C** | **GII** | **53 days** | **Con, Ilizarov** | **U** |  |
| **13.** | **47** | **M** | **3** | **T** | **22** | **1** |  | **Bl** | **IIIa** |  | **51 days** | **Ilizarov** | **Ununited** | **Ununited** |
| **14.** | **49** | **M** | **3** | **T** | **18** | **1** |  | **Co** | **C** | **GII** | **17 days** | **IL** | **U** |  |
| **15.** | **55** | **M** | **3** | **T, F,T** | **36** | **1 TA 1** |  | **L,Co,L** | **II,II,IIIa** |  | **61 days** | **Con, Ilizarov** | **U.U** | **Stiffness** |
| **16.** | **63** | **M** | **3** | **F** | **18** | **1** | **1** | **L** | **II** |  | **15 days** | **Ilizarov** | **U** |  |
| **17.** | **15** | **M** | **3** | **T** | **18** | **1** | **1** | **L** | **C** | **GI** | **14days** | **ILN** | **U** |  |
| **18.** | **19** | **M** | **3** | **T** | **27** | **1** | **1** | **L** | **I** |  | **17 days** | **ILN** | **U** |  |
| **19.** | **41** | **M** | **3** | **T** | **18** | **1** | **1** | **Co** | **II** |  | **14 days** | **Ilizarov** | **U** |  |
| **20.** | **53** | **F** | **3** | **T** | **27** | **TA** | **2** | **Co** | **C** | **GII** | **14 days** | **Ilizarov** | **U** |  |
| **21.** | **48** | **F** | **3** | **T** | **27** | **1** | **1** | **S** | **C** | **GII** | **14 days** | **ILN** | **U** |  |
| **22.** | **31** | **M** | **3** | **T** | **27** | **1** | **1** | **L** | **C** | **GII** | **19 days** | **ILN** | **U** |  |
| **23.** | **63** | **M** | **4** | **T** | **18** | **1** | **2** | **L** | **I** |  | **23 days** | **Ilizarov** | **U** |  |
| **24.** | **61** | **F** | **4** | **T** | **27** | **1** | **1** | **Bl** | **II** |  | **27 days** | **Ilizarov** | **U** |  |
| **25.** | **53** | **F** | **4** | **T** | **18** | **1** |  | **Bl** | **II** |  | **21 days** | **Ilizarov** | **Ununited** | **Ununited** |
| **26.** | **57** | **M** | **4** | **T** | **18** | **1** |  | **L** | **I** |  | **21 days** | **Ilizarov** | **U** |  |
| **27.** | **38** | **M** | **4** | **F** | **27** | **1** |  | **L** | **C** | **GII** | **29 days** | **ILN** | **U** |  |
| **28.** | **33** | **F** | **4** | **H** | **18** | **1** |  | **Co** | **II** |  | **33 days** | **Ilizarov** | **U** |  |
| **29.** | **35** | **M** | **4** | **H** | **27** | **1** |  | **Bl** | **IIIa** |  | **14 days** | **Ilizarov** | **Ununited** | **Ununited** |
| **30.** | **25** | **F** | **4** | **T,P** | **27** | **1 TA** | **1** | **L** | **C** | **GII** | **13 days** | **Con, Ilizarov** | **U** |  |
| **31.** | **26** | **F** | **4** | **T,P** | **27** | **1 TA** | **1** | **L** | **II** | **GII** | **41 days** | **Con** | **U** |  |
| **32.** | **19** | **M** | **4** | **F** | **18** | **1** | **2** | **Co** | **I** |  | **13 days** | **ILN** | **U** |  |
| **33.** | **21** | **M** | **4** | **H** | **18** | **1** | **1** | **Co** | **IIIa** |  | **12 days** | **Ilizarov** | **U** |  |
| **34.** | **27** | **F** | **4** | **T,F** | **27** | **2** | **2** | **Bl, Co** | **II, II** |  | **31 days** | **Ilizarov, ILN** | **U, U** |  |
| **35.** | **28** | **F** | **5** | **F** | **18** | **1** | **1** | **L** | **C1** | **GII** | **27 dats** | **Ilizarov** | **U** |  |
| **36.** | **29** | **M** | **5** | **F** | **18** |  |  | **L** | **C1** | **GI** | **17 days** | **ILN** | **U** |  |
| **37.** | **31** | **M** | **5** | **F, H** | **27** | **1 TA** | **1** | **L ,Co** | **II, C** | **GI** | **21 days** | **Ilizarov, ILN** | **U, U** |  |
| **38.** | **19** | **M** | **5** | **F** | **18** | **1** | **1** | **L** | **I** |  | **19 days** | **ILN** | **U** |  |
| **39.** | **25** | **M** | **5** | **F** | **27** | **1** |  | **L** | **C** | **GI** | **17 days** | **Ilizarov** | **U** |  |
| **40.** | **37** | **M** | **5** | **F** | **27** | **1** |  | **L** | **I** |  | **28 days** | **ILN** | **U** |  |
| **41.** | **33** | **F** | **5** | **F** | **27** | **1** | **2** | **Co** | **C** | **GI** | **29 days** | **ILN** | **U** |  |
| **42.** | **41** | **F** | **5** | **F, T** | **22** | **11** | **1** | **S, Co** | **II, I** |  | **31 days** | **ILN × 2** | **U, U** |  |
| **43.** | **45** | **M** | **5** | **F** | **27** | **1** |  | **L** | **C** | **GII** | **33 days** | **ILN** | **U** | **Infection** |
| **44.** | **51** | **F** | **6** | **H** | **18** | **1** |  | **Bl** | **II** |  | **17 days** | **Ilizarov** | **U** |  |
| **45.** | **53** | **M** | **6** | **H** | **18** | **1** |  | **Bl** | **I** |  | **14 days** | **Ilizarov** | **U** |  |
| **46.** | **43** | **M** | **6** | **H, T** | **27** | **1 TA** |  | **Bl, L** | **II, II** |  | **11 days** | **Ilizarov, Ilizarov** | **U, U** |  |
| **47.** | **47** | **M** | **6** | **H** | **18** | **1** |  | **L** | **IIIa** |  | **9 days** | **Ilizarov** | **U** |  |
| **48.** | **57** | **F** | **6** | **H** | **27** | **1** |  | **Co** | **II** |  | **7 days** | **Ilizarov** | **U** |  |
| **49.** | **61** | **F** | **6** | **P, T** | **27** | **1 TA** | **1** | **L** | **I** |  | **15 days** | **Con, Ilizarov** | **U, U** | **Stiffness** |
| **50.** | **63** | **F** | **6** | **P, T** | **27** | **1 TA** | **1** | **L** | **II** |  | **18 days** | **Con, Ilizarov** | **U, U** |  |
| **51.** | **62** | **F** | **6** | **P** | **27** | **1** |  | **-** | **C** |  | **21 days** | **Plating** | **U** |  |

TABLE 1

M; Male, F; Female, T; Tibia, F; Femur, P; Pelvis, H; Humerus, NISS; New injury severity score, TA; Transarticular, 1; Single bone, L; Linear, Co; comminuted, Bl; Bone loss, S; Segmental, C; Closed, Con; Conservative, U; Union,
